# Whole Genome Sequencing of Drug Resistant and Drug Susceptible *Mycobacterium tuberculosis* Isolates From Tigray Region, Ethiopia

**DOI:** 10.3389/fmicb.2021.743198

**Published:** 2021-12-06

**Authors:** Letemichael Negash Welekidan, Solomon Abebe Yimer, Eystein Skjerve, Tsehaye Asmelash Dejene, Håvard Homberset, Tone Tønjum, Ola Brynildsrud

**Affiliations:** ^1^Department of Production Animal Medicine, Norwegian University of Life Sciences, Oslo, Norway; ^2^Division of Biomedical Sciences, Department of Medical Microbiology and Immunology, College of Health Sciences, Mekelle University, Mekelle, Ethiopia; ^3^Coalition for Epidemic Preparedness Innovations, Oslo, Norway; ^4^Unit for Genome Dynamics, Department of Microbiology, University of Oslo, Oslo, Norway; ^5^Unit for Genome Dynamics, Department of Microbiology, Oslo University Hospital, Oslo, Norway; ^6^Norwegian Institute of Public Health, Oslo, Norway

**Keywords:** whole-genome sequencing, disputed *rpoB* mutations, drug resistance, *Mycobacterium tuberculosis*, Tigray, Ethiopia

## Abstract

**Background:** Tuberculosis, mainly caused by Mycobacterium tuberculosis (Mtb), is an ancient human disease that gravely affects millions of people annually. We wanted to explore the genetic diversity and lineage-specific association of Mtb with drug resistance among pulmonary tuberculosis patients.

**Methods:** Sputum samples were collected from pulmonary tuberculosis patients at six different healthcare institutions in Tigray, Ethiopia, between July 2018 and August 2019. DNA was extracted from 74 Mtb complex isolates for whole-genome sequencing (WGS). All genomes were typed and screened for mutations with known associations with antimicrobial resistance using *in silico* methods, and results were cross-verified with wet lab methods.

**Results:** Lineage (L) 4 (55.8%) was predominant, followed by L3 (41.2%); L1 (1.5%) and L2 (1.5%) occurred rarely. The most frequently detected sublineage was CAS (38.2%), followed by Ural (29.4%), and Haarlem (11.8%). The recent transmission index (RTI) was relatively low. L4 and Ural strains were more resistant than the other strains to any anti-TB drug (*P* < 0.05). The most frequent mutations to RIF, INH, EMB, SM, PZA, ETH, FLQs, and 2nd-line injectable drugs occurred at *rpoB* S450L, *katG* S315T, *embB* M306I/V, *rps*L K43R, *pncA* V139A, *ethA* M1R, *gyr*A D94G, and *rrs* A1401G, respectively. Disputed *rpoB* mutations were also shown in four (16%) of RIF-resistant isolates.

**Conclusion:** Our WGS analysis revealed the presence of diverse Mtb genotypes. The presence of a significant proportion of disputed *rpoB* mutations highlighted the need to establish a WGS facility at the regional level to monitor drug-resistant mutations. This will help control the transmission of DR-TB and ultimately contribute to the attainment of 100% DST coverage for TB patients as per the End TB strategy.

## Introduction

Tuberculosis (TB), caused by closely related Mtb complex (MTBC) species, is an ancient human disease that continues to affect millions of people every year worldwide. In 2019, there were 10.0 million incident cases and approximately 1.41 million deaths due to TB (World Health Organization, 2019). Ethiopia is among the top 30 of high TB-burden countries globally, with 157,000 incident TB cases in 2019 (World Health Organization, 2019).

Conventional genotyping and, more recently, whole-genome sequencing (WGS) indicate that MTBC is diverse globally. Different phylogenetic lineages vary markedly in their geographic distribution (Niemann and Supply, 2014).

The seven MTBC lineages are associated with the different epidemiological profiles, host range, pathogenicity, geographic regions, and drug resistance (DR) (Coscolla and Gagneux, 2014). Genomic diversity within and between MTBC lineages revealed that the most geographically widespread “generalist” L2 (Beijing), L3 (East-African-Indian), and L4 (Euro-American) are more virulent than other “specialist” lineages like L5, L6, and L7 that are more geographically restricted (Coscolla and Gagneux, 2014). According to current evidence, L7 originated in, and remains restricted to, Ethiopia, Horn of Africa (Firdessa et al., 2013). and is of significant evolutionary interest because of the phylogenetic positioning of Mtb L7 strains between the ancient L1 (Indo-Oceanic) and modern lineages L2, L3, and L4 (Nebenzahl-Guimaraes et al., 2016). Recently discovered lineages of MTBC are claimed to be restricted to the African Great Lakes region (L8) (Ngabonziza et al., 2020) and East Africa (L9) (Coscolla et al., 2021).

Phylogenetic Mtb studies have revealed that various genotypes entered Ethiopia as a consequence of human movement and trade. Moreover, TB mortality in Africa increased due to the entry of European Mtb strains and the expansion of selected native strains with a fitness benefit in the urban settings of post-colonial Africa (Comas et al., 2015).

The strategies to control TB disease are rapid and accurate diagnosis, effective treatment of active cases, and stopping transmission chains in the community (Niemann and Supply, 2014; Glaziou et al., 2018). Knowledge of the transmission dynamics of Mtb is crucial from the perspective of TB management in the community (Perdigão et al., 2017). WGS has the highest resolution for determining transmission dynamics and identifying clusters (Niemann and Supply, 2014).

Molecular studies in the capital city, Addis Ababa (Damena et al., 2019), and the northwestern (Yimer et al., 2013) parts of Ethiopia have shown extensive strain diversity. Ethiopia is a demographically complex country, and it is highly unlikely that the strain composition found in the capital would be the same as in rural regions. Hence, further studies in different regions for adequate understanding and management of TB in the country are needed (Tessema et al., 2013). No previous studies have been conducted to explore the molecular diversity and recent transmission dynamics of MTBC in the Tigray Region. Therefore, the current study aimed at assessing the genetic diversity of pulmonary TB-associated MTBC in the Tigray Region of Ethiopia using WGS.

## Materials and Methods

### Setting, Participants, and Sputum Collection and Analysis

**FIGURE 1:**
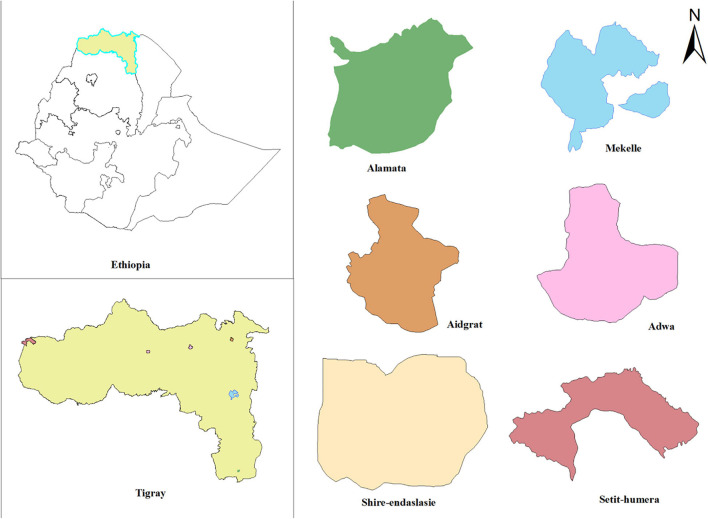
Map of the study area showing locations of hospitals in Tigray Region, Ethiopia, July 2018–August 2019.

The study was conducted in six hospitals from the six main zones of Tigray Region, northern Ethiopia (Figure 1). A hospital-based cross-sectional study was conducted from July 2018 to August 2019. Pulmonary TB patients who were not under active treatment, ≥ 15 years of age, and GeneXpert test positive were included. A 5–10 ml sputum sample was collected from eligible study participants for growth and identification of mycobacteria (Ministry of Health of Ethiopia, 2016).

### Whole-Genome Sequencing

We collected the isolates from 300 study participants who fulfilled the inclusion criteria, primarily GeneXpert positive. Among these, 227 isolates were Mtb culture positive, and the 227 Mtb culture positive were transported from Tigray Region of Ethiopia where the study was conducted to the University of Oslo, Norway where sub-culturing and DNA extraction took place. As the cost for performing WGS was very high, we selected 74 MTBC culture-positive isolates (26 MDR-TB, three mono-resistant and 45 susceptible isolates as confirmed by LPA) for WGS. In order to have a relatively representative susceptible isolates, we used defined criteria to make the selection. These included duration of illness < 60 days without hemoptysis, duration of illness ≥ 60 days with hemoptysis, imprisonment period < 60 days and imprisonment period ≥ 60 days; sputum smear grade < 3 and sputum smear grade ≥ 3. Primarily there were 38 MDR-TB and five mono-resistant isolates, and we were planning to include all of them for the WGS analysis, however, when we performed subculturing only 26 MDR and three mono-resistant TB isolates could grow and all of them were sequenced. Therefore, as we have included majority of the MDR-TB isolates and selected the mono resistant and susceptible isolates based on defined criteria, we have a strong belief that the possibility of selection bias is very much reduced even though it is difficult to eliminate it. DNA was extracted from 74 MTBC culture-positive isolates using a combination of physical and chemical methods. Briefly, into micro-centrifuge containing 25 mg glass beads, 400 μl TE buffer (10 mM Tris–HCl, 1 mM disodium EDTA) (pH 8.0) and 1 loopful colony were added. After vortexing, the solution was heated to 94°C for 20 min, then cooled down in a MagNA Lyser^TM^ cooling block (Roche) and shaken in the MagNA Lyser^TM^ for 3 × 90 s, with 30 s cooling down between cycles. The suspension was removed from the beads and used as starting material for DNA isolation using EZNA Bacterial DNA Kit (Omega Bio-tek) (Omega Bio-tek, 2017). Quantitative and qualitative measurements of the DNA were performed using Qubit^®^ 3.0 Fluorometer and NanoDrop spectrophotometers, respectively (Molecular Probes Life Technologies, 2015). DNA libraries were prepared using a Kapa DNA Library preparation kit and sequenced by Illumina-NextSeq 500, with a Paired-End read length of 2 × 75.

### Quality Assurance and Quality Control

The quality assessment of WGS data using FastQC (v0.11.8) (Andrews, 2010) in aggregation with the MultiQC (v0.4) (Ewels et al., 2016) indicated a homogenous read depth (1.7–4.4 M reads, median 3.3 M) and level of read duplication (11.0–35.3%, median 19.0%). All isolates had mean phred quality > 30 across read positions. No samples were found to have adapter contamination above the 0.1% level. Mtb isolates were verified by MASH (v2.1) (Ondov et al., 2016) and verified by kraken (v2.0.7-beta) (Wood et al., 2019) using the minikraken database (RefSeq bacteria, archaea, viruses build 2019-04). Per-sample statistics is summarized in Supplementary Tables 1, 2.

### Statistical Analysis

Reads were aligned to reference genome H37Rv NC_000962.3 (NCBI, 2020) using the snippy pipeline (v4.3.6) (Seemann, 2015). The mean read depth across the genome varied from 53.6 to 135.0, with a median of 96.8. Individual isolates were typed using the Coll et al. (2014) scheme with the program colltyper (v0.7) (Brynildsrud, 2017). Mykrobe predictor (v0.7.0) and TB-profiler V2.8.12 (accessed 2020-09-14) were used to screen and independently verify mutations involved in antimicrobial resistance (AMR), respectively (Bradley et al., 2015; Phelan et al., 2019). Snippy-core (v4.3.6) was used to obtain whole-genome alignments of all isolates. From this, known repeat regions such as PE/PPE regions, transposases, tandem repeats and BLAST self-hits were masked,^1^ and the resultant reduced alignment, masked by exclusion of sites in repetitive regions, was used to create a phylogenetic tree in FastTree (v2.1.10) (Price et al., 2010) using the GTR evolutionary model.

Additionally, variant call format files from Snippy were used to generate a visualization in Nextstrain (Hadfield et al., 2018). Repeat regions were again masked, the tree outgroup was set to isolate four, and ancestral node sequences generated at the nucleotide and protein levels for all genes. This allowed in-depth inspection of AMR emergence on branches of the phylogenetic tree. Finally, we used auspice.us to visualize these results. The recent transmission index was calculated as the number of clustered patients minus the number of clusters divided by the total number of patients (Yimer et al., 2015). In the present analysis, we consider isolates that have 12 or fewer SNPs between them to be considered as a cluster (Supplementary Table 3). Univariable logistic regression was used to identify associations between lineages, sublineages, and clusters with resistance to any anti-TB drugs.

## Results

Out of the 74 isolates subjected to WGS, 68 isolates were Mtb, three non-tuberculous mycobacteria (two *Mycobacterium simae* and one *Mycobacterium elephantis*), and three were non-Mycobacteria. The 68 Mtb isolates were used for further analysis in this paper. These isolates had been collected from participants whose median age was 30 years, ranging from 16 to 66 years. Of these, 47 (69.1%) were males and 21 (30.9%) females.

**FIGURE 2:**
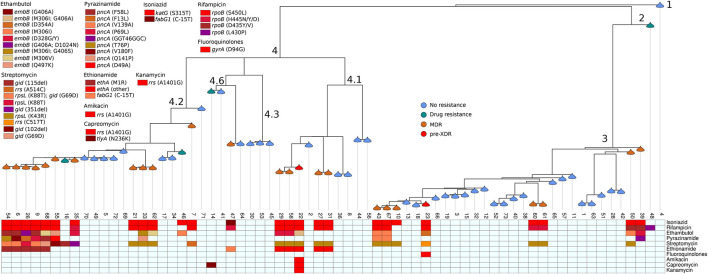
Phylogenetic tree of *Mycobacterium tuberculosis* isolated from pulmonary tuberculosis patients in Tigray Region, Ethiopia, July 2018 to August 2019. Branches are annotated with lineage, and nodes are annotated with drug resistance patterns. Boxes at the bottom part of the figure display specific anti-tuberculosis resistance-associated mutations.

The most frequent lineage was L4 (38; 55.8%), followed by L3 (28, 41.2%). Two lineages, L1 and L2, were found just once each (1.5% for each lineage). The most common sublineage circulating in the region was Central Asian Strain (CAS) (26; 38.2%) followed by Ural (20; 29.4%), Haarlem (8; 11.8%), LAM (3; 4.4%), Uganda (2; 2.9%), CAS1-Kili (2; 2.9%), and X-type (2; 2.9%). Sublineages Cameroon, T3, EAI2, Beijing, TUR, and LAM7-TUR were found just once each (1.5% for each sublineage). 15 (22%) isolates distributed into seven distinct clusters (containing two and three isolates) and 53 (78%) isolates had unique patterns, resulting in the recent transmission index (RTI) of 11.8%. Among the pre-extensively drug-resistant tuberculosis (Pre-XDR-TB), multidrug-resistant tuberculosis (MDR-TB), and isolates resistant to any 1st- and 2nd-line anti-TB drug, 100% (1/1), 91.3% (21/23), and 86.2% (25/29), respectively, distributed within the four major sublineages. Any DR to 1st- and 2nd-line anti-TB drugs was associated with L4 (OR = 2.8, 95% CI = 0.98–7.84) compared to L3 and specifically Ural strain (OR = 3.8, 95% CI = 1.11–13.46) compared to the strains identified (Table 1 and Figure 2).

**TABLE 1:** Frequency and DR association with the *Mycobacterium tuberculosis* strains and clustering status.

Variable	N (%)	Pan susceptible, F (%)	Any DR, F (%)	COR (95% Cl)	*P*-value
**Lineage**					
L3	28 (41.1)	20 (71.4) 18 (47.4)	8 (28.6) 20 (52.6)	1 2.8 (0.98–7.84)	
L4	38 (55.9)				**0.054**
**Sublineage**					
CAS	26 (38.2)	18 (69.2)	8 (30.8)	1	
Ural	19 (27.9)	7 (36.8)	12 (63.2)	3.86 (1.11–13.46)	**0.034**
Haarlem	8 (11.8)	3 (37.5)	5 (62.5)	3.75 (0.72–19.64)	0.118
LAM	3 (4.4)	3 (100)	−	−	−
L4.6/Uganda	2 (2.9)	1 (50)	1 (50)	2.25 (0.12–40.65)	0.58
CAS1-kili	2 (2.9)	2 (100)	−	−	−
X-type	2 (2.9)	2 (100)	−	−	−
L4.2.2.1/LAM7-TUR	1 (1.5)	−	1 (100)	−	
Cameroon	1 (1.5)	1 (100)	−	−	−
L4.6.2/T3	1 (1.5)	−	1 (100)	−	−
EAI2	1 (1.5)	1 (100)	−	−	−
Beijing	1 (1.5)	−	1 (100)	−	−
TUR	1 (1.5)	1 (100)	−	−	−
**Clustering**					
No	6 (8.8)	3 (50)	3 (50)	1	
Yes	62 (91.2)	36 (58.1)	26 (41.9)	0.72 (0.13–3.87)	0.7

*N, number; F, frequency; COR, crud odds ratio; DR, drug resistance. Bold values means statistically significant values.*

### Geographical Distribution of Mycobacterium Tuberculosis Lineages

**FIGURE 3:**
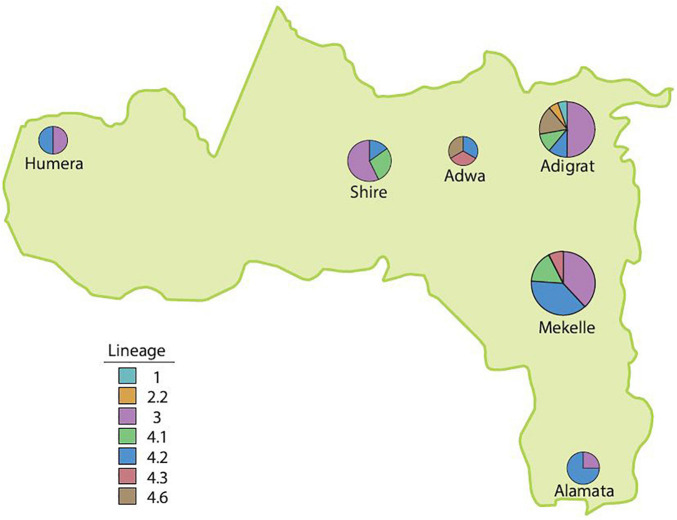
The distribution of lineages in the Tigray Region, Ethiopia, July 2018–August 2019.

The Mtb lineage distribution showed that in Alamata hospital, L4 (3; 75%; 95% CI = 22–99%) was the dominant strain, followed by L3 (1; 25%; 95% CI = 1–78%). Similarly, in Mekelle hospital, L4 (21; 61.8%; 95% CI = 44–77%) was the dominant strain, followed by L3 (13; 38.2%; 95% CI = 23–56%), and in Adwa, L4 (3; 100%; 95% CI = 31–100%) was the only strain isolated. L3 (9; 50%; 95% CI = 29–71%), L4 (7; 38.8%; 95% CI = 18–64%), L1 (1; 5.6%; 95% CI = 0.3–30%), and L2 (1; 5.6%; 95% CI = 0.3–30%) were found in Adigrat hospital. In Shire hospital, L3 (4; 57%; 95% CI = 20–88%) was the dominant strain, followed by L4 (3; 42.9%; 95% CI = 12–80%), and in Humera hospital, an equal distribution of L3 (1; 50%; 95% CI = 9–91%) and L4 (1; 50%; 95% CI 9–91%) was observed (Table 2 and Figure 3).

**TABLE 2:** Distribution of *Mycobacterium tuberculosis* strains isolated from PTB patients, Tigray Region Ethiopia, July 2018–August 2019.

Study site (hospitals)	N (%)	DR profile	L1, F (%)	L2, F (%)	L3, F (%)	L4, F (%)	Total, F (%)
Alamata	4 (5.9)	Pre-XDR	−	−	−	−	−
		MDR	−	−	−	2 (50)	2 (50)
		DR				1 (25)	1 (25)
		Pan susceptible	−	−	1 (25)	−	1 (25)
Mekelle	34 (50)	Pre-XDR	−	−	1 (2.9)	−	1 (2.9)
		MDR	−	−	3 (8.8)	11 (32.4)	14 (41.2)
		DR			1 (2.9)	−	1 (2.9)
		Pan susceptible	−	−	8 (23.5)	10 (29.4)	1 (52.9)
Adigrat	18 (26.5)	Pre-XDR	−	−	−	−	−
		MDR	−	−	1 (5.6)	1 (5.6)	2 (11.1)
		DR		1 (5.6)		1 (5.6)	2 (11.1)
		Pan susceptible	1 (5.6)	−	8 (44.4)	5 (27.8)	14 (77.8)
Adwa	3 (4.4)	Pre-XDR	−	−	−	−	−
		MDR	−	−	−	1 (33.3)	1 (33.3)
		DR				2 (66.7)	2 (66.7)
		Pan susceptible	−	−	−	−	−
Shire	7 (10.3)	Pre-XDR	−	−	−	−	−
		MDR	−	−	1 (14.3)	1 (14.3)	2 (28.6)
		DR				1 (14.3)	1 (14.3)
		Pan susceptible	−	−	3 (42.9)	1 (14.3)	4 (57.1)
Humera	2 (2.9)	Pre-XDR	−	−	−	−	−
		MDR	−	−	1 (50)	1 (50)	2 (100)
		DR	−	−	−	−	−
		Pan susceptible	−	−	−	−	−
Total	68 (100)		1 (1.5)	1 (1.5)	28 (41.2)	38 (55.8)	68 (100)

*N, number; F, frequency; DR, drug resistance; MDR, multi drug resistant; Pre-XDR, Pre-extensively drug resistant.*

### Genetic Determinants of Drug-Resistant Tuberculosis

Among the 68 MTBC isolates, 23 (33.8%; 95% CI = 23–46%) were MDR-TB, one (1.5%; 95% CI = 0–9%) pre-XDR, five (7.4%; 95% CI = 3–17%) resistant to one or more anti-TB drugs, and 39 (57.4%; 95% CI = 45–69%) were pan-susceptible. Of the 25 isolates resistant to rifampicin (RIF), the predominant canonical mutations occurred at *rpoB* codon S450L (18; 72%), followed by H445Y, H445D, and D435V at one (4%) isolate for each. The disputed *rpo*B mutations were occurred at *rpoB* codons H445N (2; 8%), D435Y (1; 4%), and L430P (1; 4%). With the exception of one isolate, which showed mutation at *fab*G1gene codon C-15T (1; 3.8%), the 25 isoniazid (INH) resistant isolates showed mutation at the *katG* gene codon S315T (25; 96.2%).

The resistance-conferring mutations for the 18 isolates resistant to ethambutol (EMB) occurred at *embB* gene codon M306I (4; 22.2%), D328G/Y (4; 22.2%), G406A (3; 16.7%), M306V (2; 11%), Q497K (1; 5.6%), and D354A (1; 5.6%). Double mutations were detected at *embB* gene codons M306I and G406A (1; 5.6%), G406A and D1024N (1; 5.6%), and M306I and G406S (1; 5.6%).

The most prevalent mutation for streptomycin (SM)-resistance (24 isolates) was at *rps*L gene codon K43R (14; 58%). Three (12.5%) isolates showed double mutations at both *rpsL* gene codon K88T and *gid* gene codon G69D. Additionally, SM resistance-conferring mutations were observed at *rps*L gene codon K88T, *rrs* gene codons A514C and C517T, *gid* gene codons 115del, 351del, 102del, and G69D from one (4.2%) isolate each.

Resistance-conferring mutations to pyrazinamide (PZA) resistant isolates (10 isolates) occurred at *pncA* gene codons V139A (2; 20%) and F58L, F13L, G46G, P69L, T76P, V180F, Q141P, and D49A in one isolate each (10%). One isolate (10%) showed a silent mutation at *pncA* gene codon G46G. Among the 11 isolates resistant to ethionamide (ETH), five (45.5%) isolates showed resistance-conferring mutations at *ethA* gene codon M1R, one (9%) at *fab*G1 gene codon C-15T, and five (45.5%) isolates showed mutations at other codons (1407del and 1341del).

The DR-conferring mutation for 2nd-line anti-TB drugs occurred at *gyr*A codon D94G for fluoroquinolone (FLQ)-resistance and *rrs* gene codon A1401G for 2nd-line injectable drugs (SLIDs) [amikacin (AMK), kanamycin (KAM), and capreomycin (CAP)] in one isolate for each. Additionally, a mutation at *tly*A gene codon N236K was detected in another isolate resistant to CAP (Table 3).

**TABLE 3:** Frequency of gene mutation for 1st-and 2nd-line anti-TB drugs.

Drug	Target gene	Mutation	N (%) ≥ 1 DR	N (%) MDR-TB	Pre-XDR	Total (*N*)
RIF (25)	*rpoB*	S450L	−	16 (64)	2 (8)	18 (72)
		H445N	−	2 (8)	−	2 (8)
		H445Y	−	1 (4)	−	1 (4)
		H445D	−	1 (4)	−	1 (4)
		D435Y	−	1 (4)	−	1 (4)
		D435V	−	1 (4)	−	1 (4)
		L430P	1 (4)	−	−	1 (4)
INH (26)	*katG*	S315T	2 (7.7)	21 (80.8)	2 (7.7)	25 (96.2)
	*fabG1*	C-15T	−	1 (3.8)	−	1 (3.8)
EMB (18)	*embB*	M306I	1 (5.6)	3 (16.7)	−	4 (22.1)
		D328G/Y		4 (16.7)	−	4 (22.2)
		G406A	−	3 (16.7)		3 (16.7)
		M306V	−	2 (11.1)	−	2 (11)
		Q497K	−	1 (5.6)	−	1 (5.6)
		D354A	−	−	1 (5.6)	1 (5.6)
		M306I; G406A	−	−	1 (5.6)	1 (5.6)
		G406A; D1024N	−	1 (5.6)	−	1 (5.6)
		M306I; G406S	−	1 (5.6)	−	1 (5.6)
SM (24)	*gid*	115del	1 (4.2)	−	−	1 (4.2)
		351del	−	1 (4.2)	−	1 (4.2)
		102del	−	1 (4.2)	−	1 (4.2)
		G69D	−	1 (4.2)	−	1 (4.2)
	*rpsL, gid*	K88T; G69D	−	3 (12.5)	−	3 (12.5)
	rpsL	K88T	−	1 (4.2)	−	1 (4.2)
		K43R	1 (4.2)	12 (50)	1 (4.2)	14 (58)
	*rrs*	A514C	−	1 (4.2)	−	1 (4.2)
		C517T	−	−	1 (4.2)	1 (4.2)
PZA (10)	*pncA*	V139A	−	2 (20)	−	2 (20)
		F58L	−	1 (10)	−	1 (10)
		F13L	−	1 (10)	−	1 (10)
		P69L	−	1 (10)	−	1 (10)
		G46G	−	1 (10)	−	1 (10)
		T76P	−	1 (10)	−	1 (10)
		V180F	−	1 (10)	−	1 (10)
		Q141P	−	1 (10)	−	1 (10)
		D49A	−	1 (10)	−	1 (10)
ETH (11)	*ethA*	M1R	−	5 (45.5)	−	5 (45.5)
		other	−	4 (36.4)	1 (9)	5 (45.5)
	*fab*G1	C-15T	−	1 (9)	−	1 (9)
FLQs (1)	*gyr*A	D94G	−	−	1 (100)	1 (100)
AMK (1)	*rrs*	A1401G	−	−	1 (100)	1 (100)
KAM (1)	*rrs*	A1401G	−	−	1 (100)	1 (100)
CAP (2)	*rrs*	A1401G	−	−	1 (50)	1 (50)
	*tlyA*	N236K	1 (50)	−	−	1 (50)

*N, number; DR, drug resistance; MDR, multi drug resistant; Pre-XDR, Pre-extensively drug resistant.*

## Discussion

WGS demonstrated the diverse lineage makeup of Mtb strains and their distribution in the Tigray Region, Northern Ethiopia. The geographic distribution of lineages revealed that strain diversity varied among the hospitals of the region. This difference could reflect patient-location differences (Maung et al., 2020). As whole-genome sequencing is very expensive, we could not sequence all of the Mtb isolates identified from participants attending the respective study sites (hospitals) in the study area. However, we included isolates that could represent the study area, as a whole. The geographic distribution of lineages within the Tigray Region is the highlight of Mtb strains isolated from each hospital. Hence, we put the frequencies or proportion of the lineages identified in the respective study sites or hospitals.

In the present study, the predominant lineage was L4, followed by L3. This is in agreement with a study reports from St. Peter’s TB Specialized Hospital, Ethiopia (Damena et al., 2019), Northwest Ethiopia (Yimer et al., 2013), Southern Ethiopia (Wondale et al., 2020), Central Ethiopia (Bedewi et al., 2017), and other African countries like Sudan (Khalid et al., 2016). Similarly, a nationwide review in Ethiopia showed that the most frequently isolated lineage was L4, followed by L3 and L1 (Mekonnen et al., 2019b). L4 isolates accounted for 91% of TB cases in Europe and the Americas, the Caribbean, the Middle East, and all African regions.

In the current study, EAI2/L1 was recovered from a single patient. Other study reports from Ethiopia (Bedewi et al., 2017; Wondale et al., 2020), Sudan (Khalid et al., 2016), and Tanzania (Katale et al., 2020) reported a higher proportion of L1. Likewise, a single isolate of L2/Beijing was observed in our study, which is in line with former studies conducted in different parts of Ethiopia (Tessema et al., 2013; Yimer et al., 2013), and another African country, Tanzania (Katale et al., 2020).

A nationwide review report from Ethiopia showed that L1 was relatively more common in Afar Region, L3 and L7 in Amhara Region, and L4 in Oromia Region, Southern Nations Nationalities and Peoples, Central Ethiopia, and Southeastern Ethiopia (Mekonnen et al., 2019b). L1 is commonly associated with populations living around the Indian Ocean, L3 is common in Central Asia and prevalent in East Africa, and L4 is common in European-Americans (Comas et al., 2015). The variation in the distribution of lineage could be associated with variation in the geographic region, ethnic group, age, and sex of patients (Maharjan et al., 2018).

According to the WGS analysis, the dominant sublineage was the CAS strain. This finding is consistent with previous studies from Southern Ethiopia (Wondale et al., 2020), Northwest Ethiopia (Tessema et al., 2013), and African countries like Tanzania (Katale et al., 2020). However, a study from Northwest Ethiopia (Yimer et al., 2013) reported the T (Tuscany) family. Studies in Kenya and Sudan reported CAS1-KILI (Ogaro et al., 2013) and Manu2 (Khalid et al., 2016) as the dominant strains. The CAS sublineage is dominant in the Indian subcontinent, South-East Asia, the Middle-East, and East-Africa. These molecular studies reveal that the presence of diverse strains and their transmission patterns are different across the world.

In Ethiopia, the major lineage and sublineages are highly diversified within the country. The second-largest sublineage in this study was Ural, followed by Haarlem, LAM, Uganda, CAS1-kili, X-type, LAM7-TUR, Cameroon, T3, EAI2, Beijing, and TUR. The sublineages Cameroon, Uganda, and CAS1-kili, were not reported in a previous study in Ethiopia (Wondale et al., 2020). The composition and proportion of these genetic diversities varied with time. Previous report revealed that L4 sublineage H is slightly higher in the Amhara Region, S is more frequent in Oromia; however, T is relatively lower in Benishangul Gumuz (Mekonnen et al., 2019b).

The MTBC lineages are classified as ancestral and “modern.” The genomes of the “modern” L2 (Beijing), 3 and 4 have undergone a large deletion known as TbD1. Lineages 2, 3, and 4 are associated with major TB epidemics and have higher transmission rates than L1 and Lineages 5–7. L3 has a higher anti-inflammatory phenotype than L4. “Modern” Mtb lineages induce less of an early inflammatory response than lineages 1 and 6. L6 develop DR less frequently, while L2 (Beijing) acquires DR more frequently (Yimer et al., 2020). The most geographically widespread L2 (Beijing) and L4 are more virulent than other lineages that are more geographically restricted. This increased virulence is associated with delayed or reduced pro-inflammatory host immune responses, greater severity of disease, and enhanced transmission (Coscolla and Gagneux, 2014).

Moreover, the country mapping of each sublineage’s proportion indicated that the distribution greatly differed by geographic location. Specifically, L4 sublineages Haarlem, LAM, and L4.10/PGG3 are distributed across the world and are called generalists, while specialist sublineages Ghana, Uganda, and Cameroon occur at high frequencies, but in specific regions of Africa or Asia, but are almost absent from Europe and the Americas. The X sublineage mainly occurred in the Americas and at lower proportions in Southern Africa, Asia, and Europe. L4.1/X, L4.2/Ural, and L4.4 occurred in high proportions in Asia and Africa, but were mostly absent from the Americas, and termed intermediate (Stucki et al., 2016). Possible reasons for the various geographic distributions of generalist and specialist sublineages could be intrinsic biological factors, extrinsic factors such as human migration, or both (Stucki et al., 2016). Although some genotypes already existed in the African continent before European contact, various genotypes were introduced to Ethiopia through human migration and trade (Comas et al., 2015).

Generally, in Africa, Mtb strains are more diversified geographically. Both generalist and specialist genotypes are circulating in the region, although L4 is the dominant lineage across the continent. TB transmission in Africa is characterized by both clustering and reactivation of Mtb strains (Mekonnen et al., 2019a).

TB disease can be a result of recent transmission of TB bacilli from active TB cases or reactivation of the previous infection. Clustering reflects the recent and active transmission of TB in the community. A cluster of two or more strains with similar genetic patterns is considered as recent transmissions, while non-clustered strains have different/unique genetic patterns and are considered as reactivation (Yimer et al., 2015). According to our WGS analysis, the overall clustering and RTI were 22 and 11.8%, respectively. The RTI of the present study is higher than the study report from Southern Ethiopia 3.9% (Wondale et al., 2020). However, this finding is lower than the overall clustering rate and RTI of previous study reports in other regions of Ethiopia: Northwest Ethiopia 30.3 and 45.1% (Tessema et al., 2013), central Ethiopia 79.8 and 68% (Bedewi et al., 2017), and a nationwide review in Ethiopia 41 and 29% (Mekonnen et al., 2019b), respectively. Clustering indicates ongoing transmission in the community, and the unique pattern indicates reactivation or recent introduction of Mtb strains into the geographic area. The relatively low RTI and clustering rate in the current study area show low level transmission of Mtb strains with high rate of reactivation or introduction of new strains. The RTI provides information on the status of active TB transmission and guides to take intervention to interrupt the ongoing transmission in the community. According to a population structure study report, the transmissibility differs among the Mtb lineages with L4 highest transmissible followed by L2, L3, and L1. Lineages 5–7 are more geographically restricted or have low-level transmissibility (Freschi et al., 2020). Possible reasons for variations in TB transmission status could be differences in socio-demographic status, study population, and strains (VanderWaal and Ezenwa, 2016). The other reason could be method difference; most of the studies used spoligotyping and mycobacterial interspersed repetitive units-variable number tandem repeats typing, which has lower power than WGS for transmission analysis due to the lower discriminatory power of these methods (Yimer et al., 2013; Mekonnen et al., 2019b).

Our finding revealed that 100% of Pre-XDR-TB, 91.3% of MDR-TB, and 86.2% of the isolates resistant to any 1st- and 2nd-line anti-TB drugs distributed within the four major sublinaeges (CAS, Ural, Haarlem, and LAM). L4 had three times more DR than L3, and sublineage Ural was four times more resistant to any anti-TB drugs than the other sublineages circulating in the region. A study report from Northwest Ethiopia indicated that Haarlem’s sublineage was associated with MDR-TB (Tessema et al., 2013). Earlier studies reported an association of W-Beijing with EMB and SM- DR (Toungoussova et al., 2003) and Beijing strains with MDR-TB (Maharjan et al., 2018). This variation could be due to the difference in the dominance of strains in the respective geographic area. The Mtb lineages have preferential geographic distributions. The association of DR with the Mtb strains varied greatly. The different DR-Mtb lineages were associated with different geographic areas (Maharjan et al., 2018). Previous studies reported that the distribution of DR profiles were associated with the various clinical characteristics of the patients. Our previous study in the same study area showed that history of previous TB treatment, cigarette smoking, intermittent fever, and duration of symptoms > 60 days were associated with MDR-TB (Welekidan et al., 2020). However, none of the sociodemographic and comorbidities were associated with MDR-TB. On the contrary, other studies from Ethiopia and other parts of the world reported the association of age, gender, and residence with MDR-TB (Hamusse et al., 2016; Workicho et al., 2017). A study by Surucuoglu et al. (2005) and Kim et al. (2008) showed that treatment failure and longer previous treatment duration of anti-TB drugs were associated with DR/MDR/XDR-TB. A study from Ethiopia showed the association of lineage 7 with patient delay in seeking treatment which may be related to the slower growth of lineage 7 strains compared to other members of MTBC (Yimer et al., 2015). Another study from China indicated that unfavorable treatment outcome was associated with infection with clustered Mtb strains (Xu et al., 2018). According to a global review on risk factors of MDR-TB, effect modification by geographic area was identified for several risk factors, which necessitate the assessment of risk factors of MDR-TB regionally to interrupt transmission and develop the most effective strategy for MDR-TB control (Pradipta et al., 2018).

Efficient TB control depends on early detection and successful treatment of TB cases. This is crucial to save lives and prevent transmission, particularly for MDR-TB (Bahizi et al., 2021). DR is attributable to the use of inappropriate treatment, delay in initiating the proper anti-TB regimens, and the use of less effective, and more toxic drugs that cause serious side effects to patients (World Health Organization, 2014). Therefore, conducting periodic drug-susceptibility testing (DST) is important to optimize patients’ treatment plan. DST data also helps to improve access to timely and standard chemotherapy and care to the patient. In addition, it helps the TB control programs in high burden countries to rapidly detect outbreaks and understand transmission trends, and real-time monitoring of control interventions.

Knowledge of the possible mutations that confer DR at specific drug targets is a key strategy for rapid detection of DR and containing the disease dissemination. The acquired DR mechanism of MTBC is associated with accumulation of spontaneous mutations at the *rpo*B gene, *kat*G/*inh*A gene, and *gyr*A/B genes for RIF, INH, and FLQs, respectively (Coll et al., 2015). Adequate evidence on the DR profile of Mtb isolates and the underlying factors including genetic profiles of Mtb strains that contribute to the development of DR has a pivotal role for the proper management of TB patients, which can reduce the incidence and recurrence of DR-TB in the community. As DR profiles depend on underlying factors and in turn, the underlying factors can have geographical variation, the regional information may contribute to design a tailored TB control and prevention strategies to achieve the WHO End TB Strategy targets. DST for 1st- and 2nd-line anti-TB drugs using the WHO recommended line probe assay (LPA) detected only RIF and INH, Fluoroquinolones (FLQs), and SLIDs resistance in the common resistance-conferring regions (Hain Lifescience, 2015). WGS enables rapid detection of DR to all 1st-and 2nd-line anti-TB drugs throughout the genome simultaneously, which could provide more information for clinical treatment, especially MDR-TB (Chen et al., 2019).

Resistance of Mtb strains to RIF is mainly due to canonical mutations in the hot-spot region of the *rpo*B gene (HSR*rpo*B). However, there are also disputed *rpoB* mutations that confer RIF-resistance and their occurrence is not rare (Jo et al., 2017). The association of these mutations with RIF-resistance is endorsed by WHO in the updated catalog of mutations in MTBC and their association with DR (World Health Organization, 2021). Previous study report indicated that low-level, but probably clinically relevant, mutations at the *rpoB* gene, called “disputed *rpoB* mutations,” are missed by standard phenotypic DST and WHO-recommended molecular techniques, like LPA and GeneXpert (Miotto et al., 2018). Thus, although the overall impact of these mutations depends on the frequency of their occurrence, which may vary geographically, they are associated with poor clinical outcomes to 1st-line treatment. Case reports from Jeong et al. (2018) showed that patients with the disputed *rpoB* mutation had successful outcomes on a high-dose (20 mg/kg) RIF-based regimen. To date, some countries, including Bangladesh (Van Deun et al., 2015), South Korea (Jo et al., 2017), and Kuwait (Al-Mutairi et al., 2019) have reported disputed *rpoB* mutations, with significant variations in proportion. In sub-Saharan Africa, studies reporting the occurrences of disputed *rpoB* mutations by utilizing WGS are very limited. In the current study, a significant proportion of disputed mutations (16%) that cause RIF-resistance was observed at *rpoB* codons H445N, D435Y, and L430P, which were not detected in our previous study using the WHO recommended LPA analysis (Welekidan et al., 2021). The 20% of RIF resistance-conferring mutations were unknown using LPA (Welekidan et al., 2021). The occurrence of disputed *rpoB* mutations is clinically and epidemiologically highly relevant (Van Deun et al., 2013). The high proportion of disputed *rpoB* mutations in the present study area suggests that the proportion of disputed *rpoB* mutations is high in TB/MDR-TB high-burden countries. Our findings may help clinicians in managing patients carrying isolates with disputed *rpoB* mutations to halt transmission of DR-TB and ultimately contribute to the attainment of 100% DST coverage for TB patients, as per the WHO End TB Strategy. According to the WGS analysis, 96.2% of mutations that confer INH-resistance occurred at *katG* codon S315T, which differed from our previous study that reported a 78% mutation at *katG* codon S315T and 19.5% unknown mutations using LPA (Welekidan et al., 2021).

Mutations at *emb*B, *emb*C, and *emb*A genes are responsible for EMB-resistance (Tekwu et al., 2014). However, in this study, mutations were detected at *emb*B gene only with the most prevalent codon M306I (22.2%) followed by D328G/Y (22.2%). The study reports showed that most mutations that cause EMB-resistance occurred at the *emb*B gene, particularly associated with codon 306, which suggests its potential as a surrogate marker for rapid detection of EMB-resistance (Zhao et al., 2015).

In our study, mutations that confer SM-resistance were observed at *rps*L, *gid*, and *rrs* genes. The most prevalent mutation associated with SM-resistance was at *rps*L gene codon K43R (58%). Although the proportion of mutations that confer resistance to SM at K43R varied geographically, this finding is concordant with an earlier study that reported its dominance across the world and its association with a high DR level (Nhu et al., 2012). The frequently detected mutation at K43R highlighted its importance as a surrogate marker for rapid detection of SM-resistance. Our findings indicated that certain isolates (12.5%) showed co-existing mutations at both genes, *rpsL* codon K88T and *gid* codon G69D. All the mutations that occurred at *gid* gene were detected from L4, particularly sublineage Ural, which suggests its value as a phylogenetic marker.

The current WGS analysis revealed that the resistance-conferring mutations to PZA predominantly occurred at *pncA* gene codons V139A (20%). The mutations seem to be concentrated at codons 46–76 and 139–141. Although *pncA* gene is the major mechanism of PZA-resistance, amino acid substitution varies among studies (Scorpio et al., 1997). In the present study, the presence of PZA-resistance conferring-mutation in all MDR-TB isolates could hinder its use as add-on anti-TB drugs for the treatment of MDR-TB.

ETH-resistance conferring mutations occurred at *ethA* gene codon M1R (45.5%), *fab*G1 gene codon C-15T (9%), and five (45.5%) isolates at other codons. Our finding revealed that all isolates resistant to ETH were co-resistant to INH. This finding is supported by prior reports that showed the isolation of Mtb strains co-resistant to INH and ETH from TB patients previously treated with INH but never treated with ETH (Vilchèze and Jacobs, 2014). The *fab*G1 gene codon C-15T mutation, which conferred resistance to both INH and ETH, was detected from MDR-TB. This is consistent with another study report that revealed mutation at *inh*A promoter region codon c-15t is detected in a large proportion of resistant isolates to INH and ETH (Walker et al., 2018). Moreover, the isolates that were resistant to ETH were from MDR/XDR-TB, which can pose lack of 2nd-line anti-TB drugs treatment alternative for MDR-TB.

In our previous study report, DR-conferring mutations to 2nd-line anti-TB drugs developed at *gyr*A gene codon D94G, D94Y/N, and A90V, but mutations that confer resistance to SLIDs were not reported by LPA (Welekidan et al., 2021). However, the present study showed mutations at *gyr*A gene codon D94G and mutations to SLIDs.

According to the WGS analysis, the mutation that conferred DR to AMK, KAM, and CAP was detected at *rrs* gene codon A1401G from one isolate each. Another mutation occurred at *tly*A codon N236K in one isolate that confers resistance to CAP. This agrees with the previous study report, which suggests it could be used as a surrogate marker for the high-level resistance to KAN and AMK (Nhu et al., 2012).

## Conclusion

The current study provides insight into Mtb strains circulating in the region, the mutation that confers DR to the anti-TB drugs and the association of frequently observed lineages and circulating sublineages with anti-TB drugs. This will guide for the management of TB patients and take interventions accordingly. In general, these findings can be used to scale up the present laboratory techniques in a way that can offer rapid and accurate results for the regular DR surveillance to interrupt the ongoing DR-TB transmission, proper management of MDR-TB as well as to design tailored TB control strategies in the region. The WGS analysis revealed the presence of diverse Mtb genotypes circulating in the Tigray Region. Overall, L4 was the most frequently observed Mtb genotype and was associated with the highest proportion of DR. The relatively low level of RTI indicates the high reactivation of DR-TB in the region. The study highlighted the usefulness of mutations at *rpoB*, *katG*, *embB*, *rps*L, *pncA*, *ethA*, *gyr*A, and *rrs* genes as molecular markers for the rapid detection of resistance for RIF, INH, EMB, SM, PZA, ETH, FLQs, and SLIDs, respectively. Given the observed high burden of MDR-TB and a significant proportion of disputed *rpoB* mutations, there is an urgent need to scale up rapid testing and detection of MDR/RR-TB cases and consider establishing a WGS facility at the regional level to monitor drug-resistant mutations. This will help in controlling transmission of DR-TB and contribute to attain the 100% DST coverage as per the End TB strategy.

## Data Availability Statement

The original contributions presented in the study are publicly available. This data can be found here: European Nucleotide Archive. The data has the project accession: ERP130987.

## Ethics Statement

The studies involving human participants were reviewed and approved by the Mekelle University, Ethical Review and Research Committee (ERC 1438/2018), Ministry of Science and Higher Education, Ethiopia (SHE/S.M/14.4/708/19), and Regional Committee for Medical Research Ethics in Eastern Norway (REK Øst) (2018/1118/REK sør-øst A). Written informed consent to participate in this study was provided by the participants’ legal guardian/next of kin.

## Author Contributions

LW, SY, ES, and TD conceived and designed the study. LW collected the data and drafted the manuscript. LW and HH conducted the laboratory work. OB and LW contributed verification and formal analysis of the data. LW, ES, SY, TD, TT, HH, and OB contributed to data analysis, reviewing, and editing the manuscript. ES, SY, and TD jointly supervised the study. All authors have seen and approved the final manuscript.

## Conflict of Interest

The authors declare that the research was conducted in the absence of any commercial or financial relationships that could be construed as a potential conflict of interest.

## Publisher’s Note

All claims expressed in this article are solely those of the authors and do not necessarily represent those of their affiliated organizations, or those of the publisher, the editors and the reviewers. Any product that may be evaluated in this article, or claim that may be made by its manufacturer, is not guaranteed or endorsed by the publisher.

## Footnotes

1
https://github.com/AdmiralenOla/GlobalL4scripts/blob/master/Problematic_regions_OBB.csv

## Supplementary Material

Supplementary Table 1 Whole-genome sequencing trimal excluded columns masked of *Mycobacterium tuberculosis* strains isolated from PTB patients, Tigray Region Ethiopia, July 2018 to August 2019.

Supplementary Table 2 Whole-genome sequencing QC metrics of *Mycobacterium tuberculosis* strains isolated from PTB patients, Tigray Region Ethiopia, July 2018 to August 2019.

Supplementary Table 3 Whole-genome sequencing pairwise SNP distances analysis of *Mycobacterium tuberculosis* strains isolated from PTB patients, Tigray Region Ethiopia, July 2018 to August 2019.

## Abbreviations

AMK: amikacin
CAP: capreomycin
DR-TB: drug resistant tuberculosis
DST: drug-susceptibility testing
EMB: ethambutol
ETH: ethionamide
FLQs: fluoroquinolones
INH: isoniazid
KAM: kanamycin
LPA: line probe assay
MDR-TB: multidrug resistant tuberculosis
Mtb: *Mycobacterium tuberculosis;*
MTBC: *Mycobacterium tuberculosis* complex
Pre-XDR-TB: pre-extensively drug resis
PZA: pyrazinamide
RIF: rifampicin
RTI: recent transmission index
SLIDs: 2nd-line injectable drugs
SM: streptomycin
TB: tuberculosis
WGS: whole genome sequencing
WHO: World Health Organization.
